# Early onset of sleep/wake disturbances in a progressive macaque model of Parkinson’s disease

**DOI:** 10.1038/s41598-022-22381-z

**Published:** 2022-10-19

**Authors:** Aurélie Davin, Stéphan Chabardès, Hayat Belaid, Daniel Fagret, Loic Djaileb, Yves Dauvilliers, Olivier David, Napoléon Torres-Martinez, Brigitte Piallat

**Affiliations:** 1 Univ. Grenoble Alpes, CEA, LETI, Clinatec, 38000 Grenoble, France; 2 Inserm, U1216, Grenoble Institut Neurosciences, Univ. Grenoble Alpes, 38000 Grenoble, France; 3 Department of Neurosurgery, University Hospital of Grenoble Alpes, 38000 Grenoble, France; 4 Department of Neurosurgery, Hospital Pitié-Salpêtrière, 75013 Paris, France; 5 UMR Inserm, 1039, Department Nuclear Medecine, University Hospital of Grenoble Alpes, 38000 Grenoble, France; 6 Center of Sleep Disorders, INM Inserm, Hopital Gui de Chauliac, Univ. Montpellier, Montpellier, France; 7 Inserm, INS, Institut de Neurosciences des Systèmes, Aix Marseille Univ, Marseille, France

**Keywords:** Circadian rhythms and sleep, Parkinson's disease

## Abstract

Parkinsonian patients often experience sleep/wake disturbances, which may appear at an early stage of the disease; however, these disturbances have not been fully described. To better understand the evolution of these disturbances with respect to disease progression, we aimed to characterize these clinical signs in a progressive nonhuman primate model of Parkinson's disease. Three adult macaques (*Macaca fascicularis)* were equipped with a polysomnographic telemetry system allowing the characterization of sleep/wake behavior via long-term neurophysiological recordings and underwent a modified multiple sleep latency test. Experiments were first performed in a healthy state and then during the progressive induction of a parkinsonian syndrome by intramuscular injections of low doses of MPTP. We observed an early onset of significant sleep/wake disturbances (i.e., before the appearance of motor symptoms). These disturbances resulted in (i) a disorganization of nighttime sleep with reduced deep sleep quality and (ii) an excessive daytime sleepiness characterized by sleep episodes occurring more rapidly in the morning and spreading through the middle of the day. The present study suggests that nighttime and daytime sleep/wake disturbances may appear early in the disease and should be considered in the development of biomarkers in further studies.

## Introduction

Sleep/wake disturbances (SWDs) are one of the most common non-motor symptoms (NMSs) of Parkinson’s disease (PD), with a prevalence of 60–70%, and lead to a significant decrease in patient quality of life^1,2^. Rapid-eye movement (REM) sleep behavior disorders (RBDs), insomnia and an increase in nighttime awakenings^3–5^ are frequently experienced by patients**.** Additionally, altered wakefulness is often reported, characterized by excessive daytime sleepiness (EDS)^1,6,7^. Furthermore, some patients exhibit EDS with sleep attacks, consisting of REM sleep, as in narcolepsy^6,8,9^. These signs, initially attributed to motor impairments or the effects of dopaminergic medications, illustrate complete disorganization of circadian rhythm^10^ and may instead be an intrinsic feature of the disease itself. Indeed, the degenerative process in PD mainly affects dopaminergic neurons in the substantia nigra; these changes may induce dysfunction of other brain structures involved in sleep/wake behavior, such as the noradrenergic locus coeruleus^11^, cholinergic pedunculopontine nucleus^12^ and orexin hypothalamic^13,14^ systems. Moreover, these structures also seem to be affected by neurodegeneration which may explain the worsening of SWDs over time. Interestingly, several studies have shown that SWDs, mostly RBDs and EDS, may precede motor symptoms by many years suggesting that they could be serve as biomarkers of PD^1,8,15^. At this time, RBDs are the NMSs most widely used as biomarkers of PD^15^. Additionally, multiple changes are observed during REM sleep, ranging from behavior differences to biological alterations such as changes in heart rate variability. Indeed, heart rate variability is higher in normal REM sleep than other sleep stages^16^ and seems to decrease in PD patients with RBDs^17^. The link between sleep disorders and wakefulness disorders is still controversial, as some studies have shown that nighttime sleep disorders and EDS are unrelated^6,18^, whereas others have shown that these disturbances go in parallel. In any case, the frequency and time of appearance of these specific NMSs relative to the first motor symptoms have not been fully determined. As accumulating evidence has shown that sleep is involved in clearance of metabolites from the brain^19,20^ and sleep exerts restorative and regenerative effects^21,22^, examining the evolution of SWDs could be crucial. Moreover, earlier management could improve the quality of life of PD patients and even slow disease progression.

1-Methyl-4-phenyl-1,2,3,6-tetrahydropyridine (MPTP)-treated nonhuman primates (NHPs) are recognized the model of choice for studying PD due to their similarities in brain damage and symptoms with parkinsonian patients^23,24^. Some studies have reported circadian rhythm disorders in MPTP-treated NHPs, but these findings were based on only actimetry techniques^25,26^. Very few studies using polysomnographic techniques, which provide objective analysis of the sleep/wake stages, have been conducted to describe severe nighttime sleep alterations^27–29^. Daytime sleepiness has also been reported, but its occurrence and nature have not been precisely described to date. Furthermore, none of the abovementionned studies investigated the prodromal phase (i.e. the phase that precedes the appearance of motor symptoms) instead examining only the phase with motor symptoms^25–29^. Here, we report, for the first time, the nature of nighttime alterations and daytime sleepiness in the prodromal phase, using a progressive NHP model of PD with a prodromal phase that lasted 4 to 6 months. Our results have important implications for (i) the use of chronic MPTP-treated NHPs for developing new therapeutic strategies to treat SWDs and (ii) the development of sleep/wake biomarkers that both enable earlier diagnosis and provide the opportunity to implement neuroprotective strategies.

## Results

### Chronic low doses of MPTP induce a slow onset of motor symptoms

**Figure 1:**
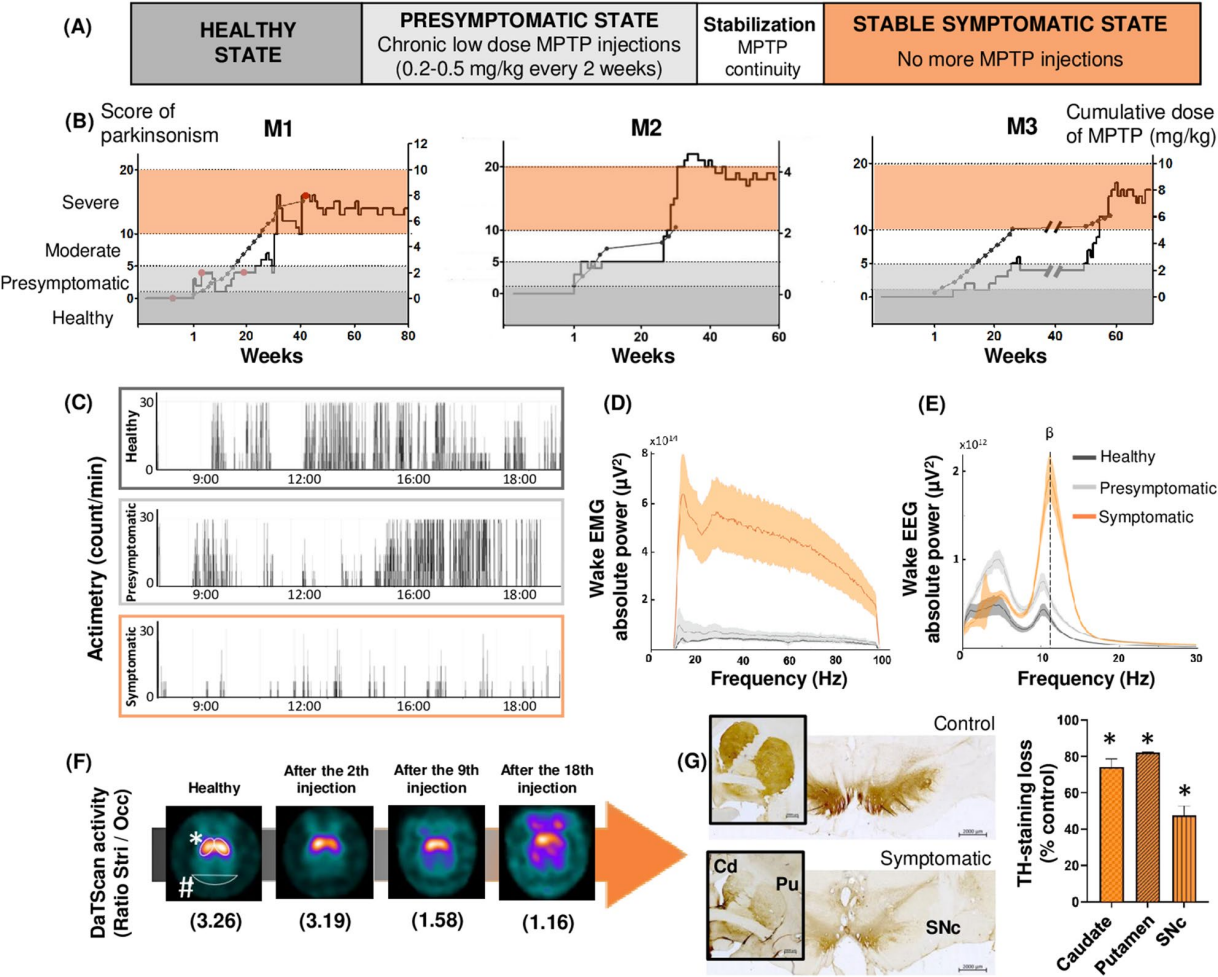
Panel of graphs showing the motor symptoms evaluation. (**A**) Schematic design study for the 3 keys periods: healthy (dark grey), presymptomatic (light grey) and symptomatic (orange) states with 8 weeks in mean of stabilization. (**B**) Longitudinal progression of Parkinson’s disease for M1, M2 and M3, induced by injection of chronic low doses of MPTP, based on weekly observations. Solid black line shows the score of parkinsonism, gray line with dot shows the cumulative dose of MPTP where each dot corresponds to an injection of 0.2–0.5 mg/kg. The red dots on M1 graph correspond to the DaTScan imaging. The line break on M3 graphs correspond to a 2-month break due to infection problems, not taken into account in analyses. (**C**) Actimetry expressed in count/min across different states of the disease from 7:00 to 19:00. (**D**) EMG spectral power density (µV^2^/Hz) during active wake across different states of the disease; healthy in dark grey, presymptomatic in light grey and symptomatic in orange, expressed in mean ± 95% confidence interval. (**E**) EEG spectral power density (µV^2^/Hz) during active wake across different states of the disease; with the appearance of low-beta activity during symptomatic state. (**F**) Study of the uptake of the Ioflupane tracer (I-123) in M1, by DaTScan nuclear imaging examination, according to the different states of the disease. * indicate regions of interest (striatum analysis area for activity measurement) and ^#^ indicate reference region (cerebral background noise measurement). Note the asymmetric pattern against the left side from the 2nd injection of MPTP, which persists with the disease progression, and the progressive increase in cerebral background noise which indicates a difficulty of tracer fixation by lack of dopamine transporters. The ratio striatal/occipital activity is in parentheses under each image. (**G**) Micrographs of control vs. symptomatic animal showing tyrosine hydroxylase (TH) immunostaining, at the level of the striatum (framed image, scale bar = 2000 µm) and the substantia nigra (scale bar = 2000 µm), associated with graph showing the mean percent loss of TH expression in the caudate (Cd), the putamen (Pu) and the substantia nigra compacta (SNc) compared to the control animal (Mann–Whitney U test, p < 0.05).

During the course of MPTP injections, animals exhibited a gradual onset of motor symptoms, allowing the identification of several key periods (Fig. 1A). First, animals went through a presymptomatic period that lasted 4 to 6 months and was characterized by the absence of visible motor symptoms; this period started just after the first MPTP injection (Fig. 1B; see Supplemental Table 1 for individual parkinsonism scores). Next, the animals exhibited a period of stabilization of the parkinsonian syndrome, characterized by the appearance of slight and unstable motor symptoms. Data collected during this fluctuating period were excluded from the analyses. Finally, animals exhibited a stable parkinsonian syndrome over several months without any signs of recovery, after an average cumulative dose of 5.2 ± 1.7 mg/kg (motor score of 16.4 ± 1.5). This parkinsonian state was characterized by a decrease in general activity (Fig. 1C), flexed posture, bradykinesia evolving into akinesia and action tremors. Three animals were included in this experiment: M1, M2 and M3. M2 experienced the most severe symptoms, induced with fewer injections of MPTP than those necessary for M1 and M3, but he was still able to perform basic self-maintenance behaviors such as eating and grooming. Compared to that in the healthy state, the spectral analysis of electromyography (EMG) data recorded during active wake had markedly higher power density during the symptomatic state and a tendency toward a higher power density during the presymptomatic state (Fig. 1D). In healthy and presymptomatic animals, spectral analysis of electroencephalography (EEG) recordings during active wake exhibited a low power density of mixed frequency. In symptomatic animals, a peak appeared in the low beta band at 13 Hz (Fig. 1E). In addition, DaTscan examinations in M1 showed a decrease in tracer uptake from the 2nd injection of MPTP, which persists and worsens with the disease progression (Fig. 1F). Note the progressive increase in the cerebral background noise after the 18th injection (corresponding to the stable symptomatic state) which indicates a difficulty of tracer fixation due to the lack of dopamine transporters. Moreover, at the end of the experiments, all three animals showed similar damage to dopaminergic neurons, with a decrease of anti-tyrosine hydroxylase (TH)-stained cells of approximately 80% in the striatum (putamen and caudate nucleus) and approximately 40% in the substantia nigra (Fig. 1G).

### Nighttime sleep disorganization: progression from the presymptomatic to symptomatic state

**Figure 2:**
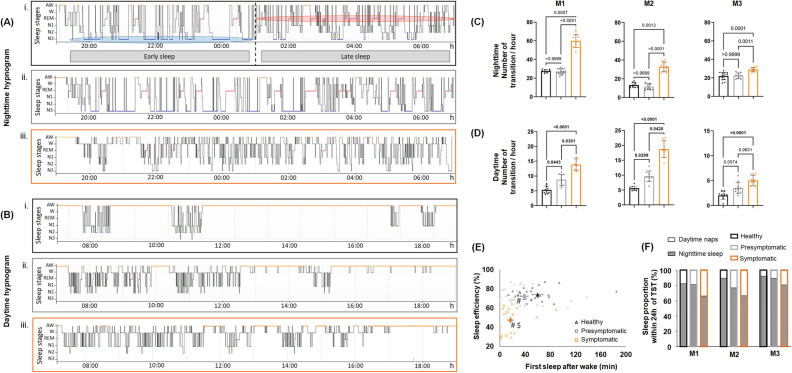
Nighttime and daytime sleep macro-architecture. (**A**) Nighttime hypnogramm (from M1), during healthy (i), presymptomatic (ii) and symptomatic (iii) states. The early sleep period is mostly composed by S3 (circled in blue) and the late sleep period is mostly composed by REM sleep (circled in red). Note the sleep fragmentation in symptomatic state where N3 and REM sleep are almost inexistent in early sleep and late sleep period respectively. (**B**) Daytime hypnogramm (from M1), during healthy (i), presymptomatic (ii) and symptomatic (iii) states. Note the naps disorganization since the presymptomatic state which is worse during symptomatic state. For both, (**A**) and (**B**) the line position indicates sleep/wake stages represented in y-axis. With A = active wake; W = quiet wake; N1, N2, N3 = non-REM sleep stage 1, 2 and 3; R = REM sleep. (**C**) and (**D**) Respectively nighttime and daytime number of transition between wake and sleep stages per hour for M1, M2 and M3, during healthy (black), presymptomatic (light grey) and symptomatic (orange) states. (**E**) Plot of nighttime sleep efficiency (%) according to the first sleep after wake (min) the following day, for all animals, during healthy, presymptomatic and symptomatic states. The mean and SEM are presented for each state. Kruskal–Wallis test followed by Dunn’s multiple comparisons test with # first sleep after wake different from healthy and $ sleep efficiency different from healthy. (**F**) Sleep proportion within 24 h of total sleep time expressed in percentage during daytime (white) and nighttime (hatched white), for M1, M2 and M3, during healthy (black), presymptomatic (light grey) and symptomatic (orange) states.

**Figure 3:**
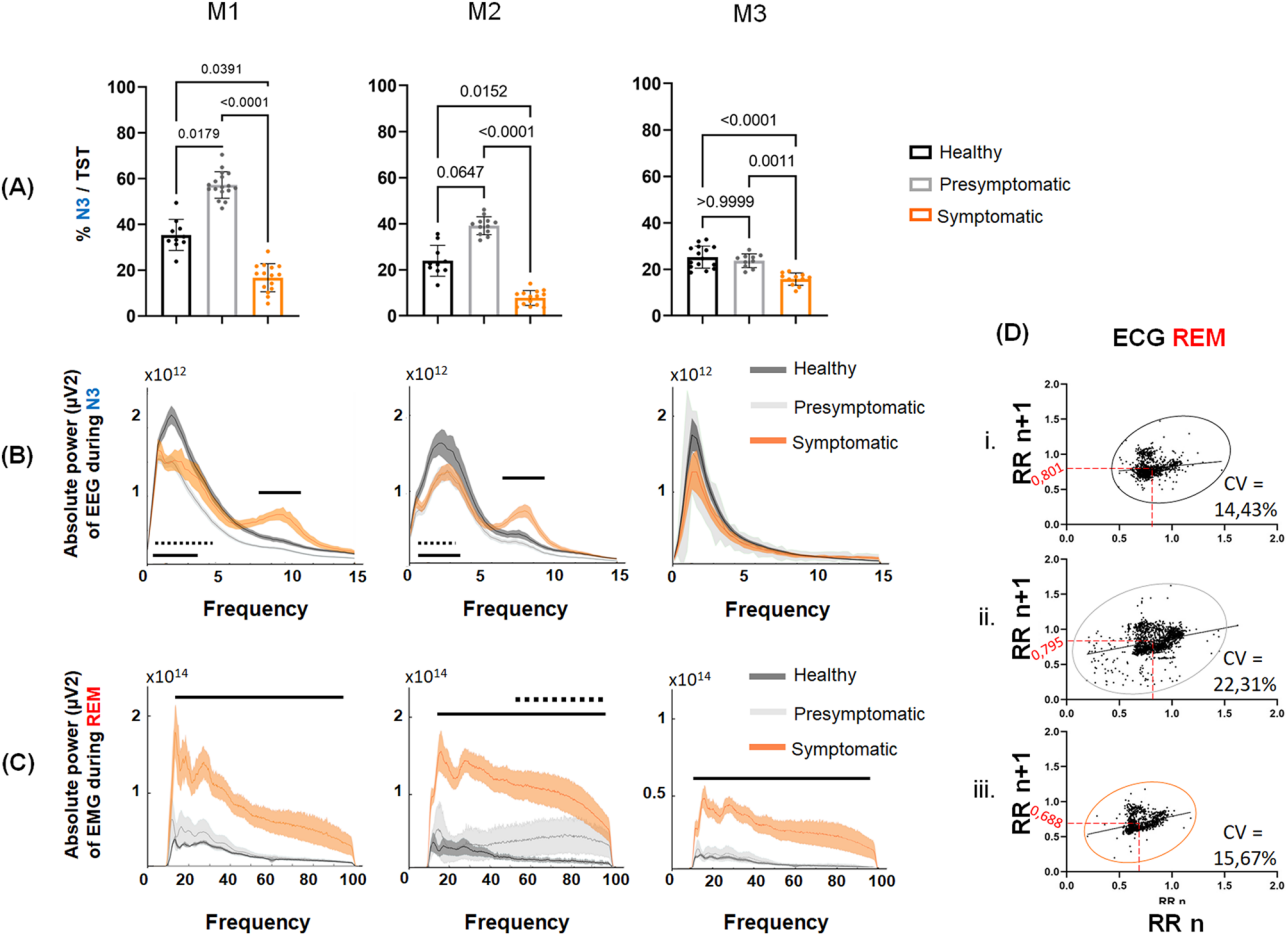
Nighttime sleep micro-architecture. (**A**) Nighttime non-REM sleep N3 quantity, for M1, M2 and M3, during healthy (black), presymptomatic (light grey) and symptomatic (orange) states, expressed as % of N3 on the TST. (**B**) Mean ± 95% confidence interval of absolute power depending on frequency of EEG during nighttime N3 for M1, M2 and M3, during healthy (dark grey), presymptomatic (light grey) and symptomatic (orange) states. (**C**) Mean ± 95% confidence interval of absolute power depending on frequency of EMG during nighttime REM sleep for M1, M2 and M3, during healthy (dark grey), presymptomatic (light grey) and symptomatic (orange) states. For (**B**) and (**C**), the dashed black line indicates significant differences between healthy and presymptomatic states and the solid black line indicates significant differences between healthy and symptomatic (one-way ANOVA; p < 0.05). (**D**) Poincare plots build from successive RR intervals (x-axis: RR n and y-axis RR n + 1) expressed in second during REM sleep during the healthy (i), presymptomatic (ii) and symptomatic (iii) states. The red number correspond to the meanNN interval and CV correspond to the coefficient of variation.

Healthy animals exhibited a similar sleep architecture with a high proportion of N3 sleep in the first half of the night and a greater proportion of REM sleep in the second half of the night (Fig. 2A). Healthy animals experienced an average of eleven well-defined sleep cycles; this number began to decrease during the presymptomatic state in M2 and M3 and was significantly decreased in all three animals during the symptomatic state (Table 1A). In the presymptomatic state, N3 accounted for a greater proportion of sleep in M1 and M2 (M1, healthy: 35.4 ± 2.1% vs. presymptomatic: 57.2 ± 1.4%; p = 0.0179 and M2, healthy: 23.9 ± 2.1%: vs. presymptomatic: 39.1 ± 1.1%; p = 0.0647) (Fig. 3A). For both animals, changes in EEG power density were observed in the delta frequency band (0.3–4 Hz) during N3, i.e., deep sleep. Indeed, during the presymptomatic state, there was a significant decrease in the power density of the delta band despite an increase in its quantity compared to the healthy state (Fig. 3B). M1 and M2 did not exhibit significant changes in the total sleep time (TST) and wake after sleep onset (WASO) and thus no changes in sleep efficiency. However, M2 showed a significant decrease in REM sleep during this period (healthy: 11.4 ± 0.7% vs. presymptomatic: 4.5 ± 0.8%; p = 0.0007). M3 did not exhibit any increase in N3 during the presymptomatic state but exhibited significant alterations in sleep parameters, such as a decrease in TST and an increase in WASO, without significant alterations in sleep efficiency (Table 1A).

**Table 1:** Sleep parameters from long-term 12 h recordings of nighttime and daytime for each animal during healthy, presymptomatic and symptomatic states.

(A)	Nighttime								
	M1			M2			M3		
	Healthy (n = 10)	Presymptomatic (n = 16)	Symptomatic (n = 15)	Healthy (n = 10)	Presymptomatic (n = 12)	Symptomatic (n = 14)	Healthy (n = 14)	Presymptomatic (n = 10)	Symptomatic (n = 17)
SL (min)	13.4 ± 1.5	22.2 ± 1.5*	18.7 ± 2.8	7.5 ± 1.2	0.3 ± 0.3*	4.8 ± 1.5	10.2 ± 1.6	17.3 ± 2.1	26.1 ± 2.1*
% AW	17.2 ± 0.8	19.0 ± 0.6	38.0 ± 1.3*^#^	15.5 ± 1	12.8 ± 0.9	28.0 ± 2.1*^#^	20.2 ± 0.8	21.7 ± 1.1	25.3 ± 1.1*
% W	10.0 ± 0.8	11.8 ± 0.6	29.0 ± 1.2*^#^	2.7 ± 0.5	2.9 ± 0.8	13.6 ± 2.3*^#^	8.8 ± 0.5	13.8 ± 0.9*	14.8 ± 1.3*
% N1	18.2 ± 1.0	10.2 ± 0.5*	13.3 ± 1.1*^#^	20.0 ± 2.0	13.8 ± 2.0	31.5 ± 0.9*^#^	16.9 ± 1.6	22.1 ± 1.5	23.6 ± 1.5
% N2	17.0 ± 2.0	5.2 ± 0.5*	8.2 ± 0.4*^#^	32.1 ± 2.2	32.2 ± 1.6	14.6 ± 2.0*^#^	24.0 ± 1.3	17.7 ± 1.3*	18.1 ± 1.2
% N3	25.2 ± 1.5	39.6 ± 1.1*	5.4 ± 0.6*^#^	18.2 ± 1.7	34.0 ± 2.3*	5.1 ± 0.9*^#^	18.6 ± 1.2	15.2 ± 1.3	8.6 ± 1.3*
% REM sleep	12.4 ± 0.4	14.3 ± 0.6	5.1 ± 0.4*^#^	11.4 ± 0.7	4.5 ± 0.8*	7.1 ± 0.7*^#^	10.7 ± 0.4	9.5 ± 0.5	9.4 ± 0.8
TST (min)	523.1 ± 5.1	498.8 ± 6.3	230.4 ± 7.0*^#^	589.4 ± 9.3	607.3 ± 11.4	419.9 ± 14.0*^#^	505.2 ± 7.9	468.4 ± 10.3*	431.3 ± 22.1*
WASO (min)	182.3 ± 5.2	197.4 ± 6.2	465.8 ± 6.7*^#^	120.1 ± 9.3	106.6 ± 11.6	289.4 ± 13.3*^#^	199.8 ± 8.0	234.9 ± 6.3*	260.9 ± 11.8*
Sleep efficiency (%)	74.2 ± 0.7	71.8 ± 0.9	33.4 ± 0.9*^#^	83.1 ± 1.3	86.2 ± 0.9	60.5 ± 1.7*^#^	71.5 ± 1.4	66.1 ± 1.5	61.9 ± 2.3*
Sleep cycle number	11.4 ± 0.3	10.3 ± 0.4	0.8 ± 0.4*^#^	11.4 ± 0.6	6.8 ± 0.9*	5.7 ± 0.7*	10.6 ± 0.3	8.7 ± 0.3*	6.6 ± 0.9*

(B)	Daytime								
	M1			M2			M3		
	Healthy (n = 10)	Presymptomatic (n = 12)	Symptomatic (n = 11)	Healthy (n = 10)	Presymptomatic (n = 10)	Symptomatic (n = 14)	Healthy (n = 10)	Presymptomatic (n = 12)	Symptomatic (n = 10)
SL (min)	41.1 ± 6.1	25.8 ± 4.8	8.7 ± 2.6*	55.0 ± 7.2	28.6 ± 5.2	16.6 ± 6.4*	77.3 ± 17.8	67.8 ± 16.3	30.4 ± 11.9
% AW	80.6 ± 1.4	76.8 ± 2.2	50.5 ± 2.4*^#^	85.8 ± 1.8	69.9 ± 3.4*	61.6 ± 2.0*	89.3 ± 1.5	86.8 ± 1.9	74.9 ± 1.9*^#^
% W	4.4 ± 0.6	7.6 ± 1.3	27.3 ± 1.7*^#^	5.3 ± 0.8	5.6 ± 0.7	9.7 ± 1.1*^#^	5.2 ± 0.7	5.9 ± 0.9	11.3 ± 0.9*^#^
% N1	8.6 ± 1.1	6.6 ± 0.5	**11.0 ± 0.8**^#^	6.0 ± 0.9	12.3 ± 2.0	18.4 ± 1.2*	3.0 ± 0.4	4.3 ± 0.7	7.6 ± 0.8*^#^
% N2	5.0 ± 0.4	4.2 ± 0.7	3.7 ± 0.5	2.9 ± 0.6	10.9 ± 2.3*	9.1 ± 2.0*	2.3 ± 0.4	2.5 ± 0.5	5.5 ± 1.2*^#^
% N3	1.1 ± 0.3	**3.2 ± 0.5** *	**0.2 ± 0.1**^#^	0.0 ± 0.0	1.3 ± 0.7*	0.1 ± 0.05	0.2 ± 0.2	0.4 ± 0.2	0.0 ± 0.0
% REM sleep	0.2 ± 0.1	**1.5 ± 0.4** *	1.2 ± 0.3*	0.1 ± 0.1	0.1 ± 0.1	1.0 ± 0.2*^#^	0.1 ± 0.1	0.1 ± 0.1	0.7 ± 0.4*
TST (min)	107.4 ± 8.2	112.1 ± 10.2	115.0 ± 7.7	64.8 ± 8.1	176.3 ± 24.3*	205.6 ± 17.7*	39.7 ± 6.1	52.6 ± 8.0	99.3 ± 12.2*^#^

(**A**) Each column represents mean values (± SEM) derived from healthy, presymptomatic and symptomatic nights (n are indicated in parentheses) for M1, M2 and M3. SL correspond to the time in minute between lights turned off (7 pm) and the first sleep episode. Values in lines 2, 3, 4 and 5 are expressed as the mean percentage of the total scoring time with AW = active wake, W = quiet wake, N1, N2, N3 = Non-REM sleep stage 1, 2 and 3 respectively and REM sleep. TST refers to the total sleep time during the night i.e. between 7 p.m. and 7 a.m. Wake time after sleep onset (WASO) is expressed in minute and refers to the sleep period time minus the TST. Sleep efficiency, expressed in %, as the ratio of TST to the sleep period time. (**B**) Each column represents mean values (± SEM) derived from healthy, presymptomatic and symptomatic days (n are indicated in parentheses) for M1, M2 and M3. Sleep latency (SL) refer to the first sleep after wake and TST refer to the total sleep time during the day. Each sleep and wake stages are expressed in mean percentage of the total scoring time. *p < 0.05: Kruskal–Wallis test followed by Dunn’s multiple comparisons test in the event of statistically significant differences.

Significant values are in bold.

Once parkinsonian syndrome was established, all animals showed strong disorganization of sleep architecture, qualitatively illustrated on hypnograms (Fig. 2A) and quantitatively indicated by a significant increase in the number of hourly transitions between wakefulness and sleep in the three animals (Fig. 2C). Additionally, we observed that the animals experienced lighter sleep since they spent less time in N3 (Fig. 3A) and more time in N1 sleep (Table 1A). In addition, changes in EEG power density during N3 were observed in M1 and M2; specifically, a significant decrease in 0.3–4 Hz power and a significant increase in 8–12 Hz power (Fig. 3B). These changes were accompanied by a significant decrease in TST associated with an increase in WASO, which led to a significant decrease in sleep efficiency in the three animals (Table 1A). Moreover, in addition to a significant disruption of REM sleep (Table 1A), the spectral analysis of the EMG during this specific sleep stage showed a progressive increase in the amplitude of muscle tone from the healthy to symptomatic state in the three animals (Fig. 3C). Consistent with these findings, examination of heart rate variability during REM sleep showed that the interval interbeat (meanNN) remained stable between the healthy and presymptomatic states but decreased significantly in the symptomatic state (0.801 ± 0.116 s vs. 0.795 ± 0.177 s vs. 0.688 ± 0.108 s, respectively). However, we observed an increase in the variability of beat-to-beat RR in the presymptomatic state characterized by a higher coefficient of variation than in the healthy and symptomatic states (healthy: 14.43% vs. presymptomatic: 22.31% vs. symptomatic: 15.67%) (Fig. 3D).

### Daytime hypersomnia assessment: progression from the presymptomatic to symptomatic state

Healthy animals exhibited three to four naps during the day lasted 10 to 60 min each. From the presymptomatic to symptomatic states, wakefulness became increasingly fragmented by sleep episodes throughout the day, qualitatively illustrated on hypnograms (Fig. 2B) and quantitatively indicated by the significant increase in hourly transitions between wake and sleep stages (Fig. 2D). Animals’ first nap after wake occurred earlier with progression of the parkinsonian syndrome and occurred significantly earlier during the symptomatic state for M1 and M2 (M3 displayed similar tendency) (Table 1B). Interestingly, we observed that the timing of first nap after waking significantly changed even before alterations in nighttime sleep efficiency. Indeed, during the presymptomatic state, we observed significantly shorter sleep latencies in the morning even though sleep efficiency remained stable. During the symptomatic state, we observed even shorter sleep latencies in the morning associated with a decrease in sleep efficiency for all animals (Fig. 2E). In all animals, the nap architecture changed over the course of disease progression; with many inter-individual variations (Table 1B). In M1 and M2, during the presymptomatic state, we observed an increase in N3 compared to that during the healthy state; this increase which was almost entirely abolished during the symptomatic state (Table 1B). M1 exhibited significant increases in REM sleep compared to that in the healthy state over the course of disease progression (healthy: 0.2 ± 0.1% vs. presymptomatic: 1.5 ± 0.4%; p = 0.0120 vs. symptomatic: 1.2 ± 0.3%; p = 0.0340). All three animals exhibited an increase in N1 during daytime naps. M2 and M3 exhibited a progressive change in the proportion of nighttime sleep and daytime naps in 24 h TST. Indeed, these two animals slept more during the day and less during the night with disease progression. The same trajectory was observed in M1 but only during the symptomatic state (Fig. 2F).

### Early onset of daytime sleepiness on the modified multiple sleep latency test

**Figure 4:**
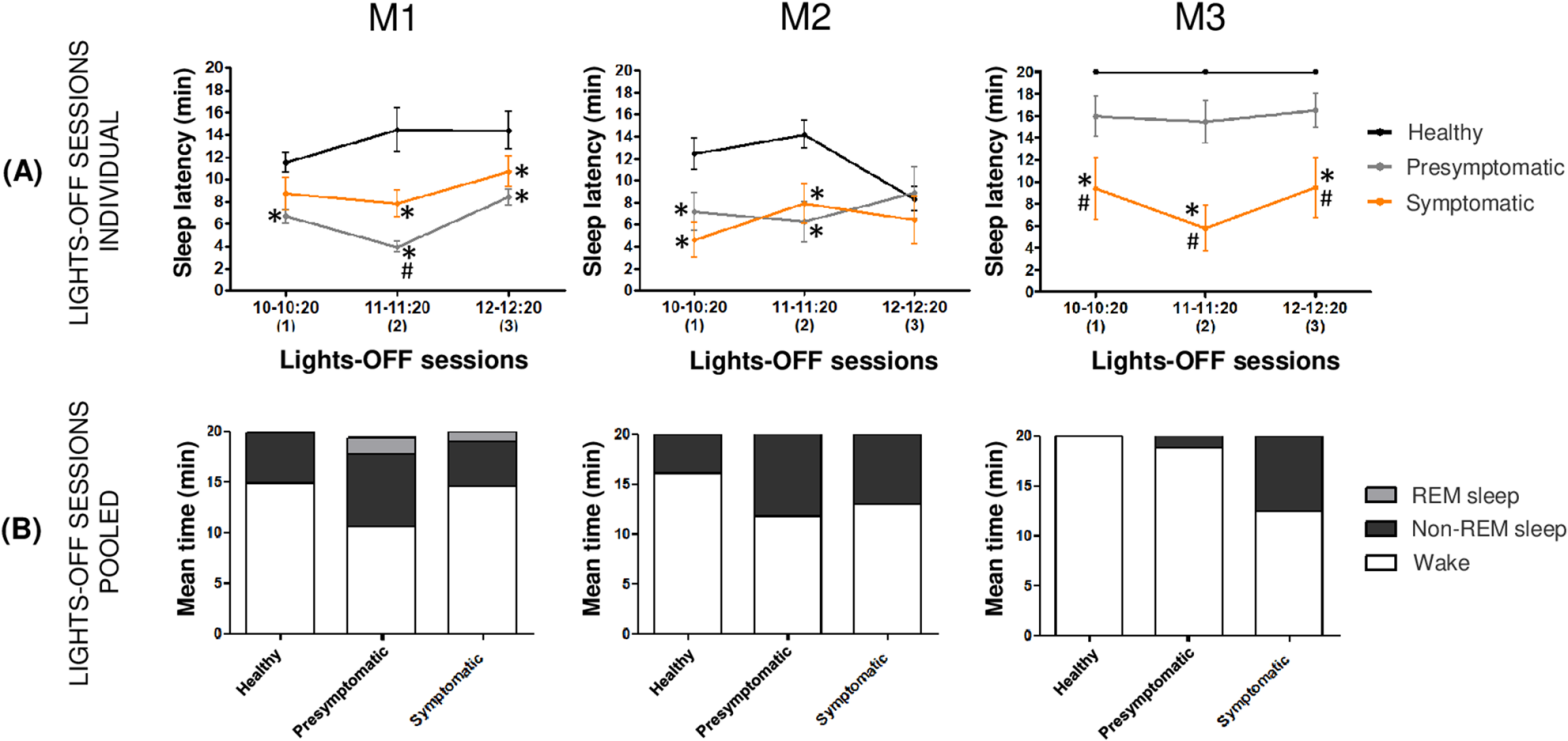
Panel of different sleep parameters obtained in lights-OFF sessions of each animal during healthy, presymptomatic and symptomatic states. (**A**) SL expressed in min for each lights-OFF session (1) from 10 a.m. to 10:20 a.m., (2) from 11 a.m. to 11:20 a.m., (3) from 12 a.m. to 12:20 a.m. and during healthy (black line), presymptomatic (grey line) and symptomatic (orange line) states. (**B**) Mean time of wake (white), non-REM sleep (N1-N2-N3) (black) and REM sleep (grey) duration for all lights-OFF session pooled together during healthy, presymptomatic and symptomatic states. P < 0.05 * different from healthy state; # different from presymptomatic state: Kruskal–Wallis test followed by Dunn’s multiple comparisons test in the event of statistically significant differences.

During the presymptomatic state, the modified multiple sleep latency test (mMSLT) showed that M1 and M2 fell asleep significantly more rapidly than they did in the healthy state (M1, healthy: sleep latency (SL): 13.5 ± 0.9 min vs. presymptomatic: 6.3 ± 0.4 min and M2, healthy: 11.6 ± 0.8 min vs. presymptomatic: 7.4 ± 1.1 min), and M3 exhibited some sleep episodes for the first time (SL: 16 ± 1 min) (Table 2). This phenomenon was observed since the first MPTP injection. During the presymptomatic and symptomatic states, the SL was shorter than it was in the healthy state for each lights-OFF session (mean example of SL in lights-OFF session 1 for M2, healthy: 12.43 ± 1.45 min vs. presymptomatic: 7.17 ± 1.73 min vs symptomatic: 4.61 ± 1.59 min) except lights-OFF session 3 for M2 (Fig. 4A). M1 and M2 presented a significant increase in nap duration during the presymptomatic and symptomatic states compared to the healthy state (Fig. 4B). M3 did not exhibit substantial napping during the presymptomatic state (1.5 ± 0.5 min) but exhibited a strong increase in nap duration during the symptomatic state (7.6 ± 1.3 min). For all monkeys, naps occurring during the lights-OFF sessions mainly consisted of N1-N2 sleep. Interestingly, M1 exhibited a few episodes of N3 and REM sleep during the presymptomatic state and symptomatic states (Fig. 4B).

**Table 2:** Sleep parameters obtained in lights-OFF sessions for each animal M1, M2 and M3 during healthy, presymptomatic and symptomatic states.

	**M1**			**M2**			**M3**		
	Healthy (n = 30)	Presymptomatic (n = 120)	Symptomatic (n = 90)	Healthy (n = 30)	Presymptomatic (n = 48)	Symptomatic (n = 39)	Healthy (n = 30)	Presymptomatic (n = 57)	Symptomatic (n = 39)
SL (min)	13.5 ± 0.9	6.3 ± 0.4*	9.8 ± 0.8*^#^	11.6 ± 0.8	7.4 ± 1.1*	6.3 ± 1.1*	20	16 ± 1	8.2 ± 1.4*^#^
N1–N2 (min)	5.1 ± 0.7	7.2 ± 0.3*	4.3 ± 0.6	3.9 ± 0.5	8.2 ± 0.9*	7 ± 0.8*	0	1.5 ± 0.5	6.7 ± 1.2*^#^
N3 (min)	0	0.6 ± 0.2*	0.1 ± 0.1	0	0	0	0	0	0
REM Sleep (min)	0	1.6 ± 0.2*	1 ± 0.3*	0	0	0	0	0	0

Sleep parameters obtained in lights-OFF sessions for each animal during healthy, presymptomatic and symptomatic states expressed in mean values (± SEM) derived from lights-OFF sessions pooled together for each condition and animal (n are indicated in parentheses). SL refer to the time in minute between the lights turned off and the first sleep episode. Values in lines 2, 3 and 4 are expressed as the mean duration ± SEM in min for each sleep stages (N1 and N2 pooled together, N3 and REM sleep). P < 0.05 * different from healthy state; ^#^different from presymptomatic state: Kruskal–Wallis test followed by Dunn’s multiple comparisons test in the event of statistically significant differences.

Significant values are in bold.

## Discussion

In this study, we described SWDs in a progressive NHP model of PD. We showed that NHPs exhibited some SWDs before the appearance of motor symptoms, such as disorganization of nighttime N3 sleep which become lighter and EDS characterized by sleep episodes occurring more rapidly in the morning and spreading through the middle of the day, leading to fragmented wakefulness. These disturbances worsened with disease progression, producing a drastic decrease in nighttime sleep efficiency and persistent EDS. In parkinsonian patients, these SWDs are also reported^3,7^, but the precise chronology of their appearance compared to that of motor symptoms has not been fully documented. Nevertheless, it seems that SWDs may appear at an early stage of the disease, indicating a possible prodromal phase^1^. The use of NHPs in this study is justified by the fact that (1) they exhibit sleep/wake behavior similar to that in humans^30,31^ and (2) MPTP injections can induce a parkinsonian syndrome in NHPs similar to that in human patients^27,28^.

The progressive induction used in this study allowed us to reproduce the slow progression of PD with a presymptomatic state (lasting 4–6 months) characterized by no significant motor symptoms and no beta oscillations on the EEG. This feature is in accordance with another NHP study that showed that beta oscillations are correlated with the severity of PD and are significantly increased once a parkinsonian syndrome is induced^32^. This phenomenon is also reported in human patients and seems to be both causally and quantitatively related to the PD-induced motor impairment^33^. Based on these findings, this presymptomatic period in NHPs can be considered similar to the presymptomatic state encountered in human PD patients. After this presymptomatic period, all three animals developed a stable and similar parkinsonian syndrome that lasted for months, during which time they did not exhibit signs of recovery. Compensation phenomena take place very quickly in a more acute MPTP model^34^ (i.e. administration of a higher dose of MPTP over a shorter period of time); therefore, the model used in our study provides stability and the possibility of studying long-term parameters. Importantly, M2 exhibited a more severe parkinsonian syndrome than the other two animals after fewer MPTP injections. This difference may be explained by the fact that he was the oldest animal in the study. Indeed, studies have shown that the sensitivity to MPTP increases with age, and our findings are entirely consistent with this observation^35^.

During the presymptomatic state, sleep quality was significantly altered, with an increase in nocturnal awakenings in one animal (M3) and with a less intense but longer N3 stage in two of three animals (M1 and M2). This increase in N3 was reported in another MPTP-NHPs study, but the changes in EEG delta power was not described^28^. Interestingly, N3 has long been considered a restorative sleep stage^36,37^ and its disruption appears to be closely linked to the progression of neurodegenerative diseases such as Alzheimer’s disease and more recently to PD^38^. As N3 appears to be important for the brain clearance, in a physiological but also pathological context^39,40^, additional N3 durations could reflect adaptive processes activated in the early stage of the disease to counteract the decrease in delta power and the disease progression itself. We also found an increase in the 8–12 Hz frequency during N3; this increase could be an indirect effect of the reduced delta power that, therefore, could be counteracted by faster activity. Interestingly, this frequency band is characteristic of slow sleep spindles^41^. Sleep spindles are bursts of oscillatory brain activity that originate in the thalamus and play a role in sleep maintenance^42^. Some studies have demonstrated a positive correlation between spindle density and the duration or stability of sleep^43^, suggesting that the amount of sleep spindles might signify the strength of the gatekeeping mechanism that the thalamus exerts during sleep. Additionally, a causal role for sleep spindles in regulating sleep was established by a study showing that optogenetic enhancement of spindle-like rhythmicity produced a direct stabilizing effect on non-REM sleep duration, supporting the protective action of sleep spindles^44^. Therefore, the induction of slow sleep spindles during N3 can be regarded as an additional compensatory mechanism to protect sleep elicited by delta power loss. Current studies on sleep spindles during the N3 sleep stage in patients with PD are scarce and not all agree, some have shown a decrease in their density^45^, while others have shown no change^46^. It would be interesting to perform further studies on spindle characteristics especially during the presymptomatic state to better understand their role. In addition, the EMG power tended to increase during REM sleep in the presymptomatic state; this loss of atonia may also be a premotor sign of PD. Paralleling our REM sleep findings, we also showed a significant increase in heart rate variability during the presymptomatic state, characterized by an increase in the coefficient of variation of RR intervals with a similar mean heart rate. These significant changes provide interesting prodromal tools for further analyses.

Additionally, several specific daytime disturbances were identified. All animals exhibited sleep inertia (i.e. difficulties waking up in the morning), resulting in a first nap shortly after waking and continuous naps until the middle of the day. This altered wakefulness was also revealed by mMSLT results, which showed shorter SL and longer sleep episodes. These SWDs were significant in M1 and M2 and less marked in M3. Nevertheless, M3 experienced sleep episodes during the lights-OFF sessions of the mMSLT for the first time, highlighting a real impairment of wakefulness. The fact that M3 was younger than M1 and M2 can explain these differences. In addition, we highlight the occurrence of REM sleep episodes in M1 during lights-OFF sessions in the presymptomatic state; this phenomenon, typical of sleep attacks encountered in narcolepsy, has also been described in human PD patients. Moreover, the discrepancies among our animals are consistent with the variability of PD symptoms observed in humans^47^. Our results also showed that EDS correlated with poor nighttime sleep quality, suggesting that changes in sleep and wakefulness may go hand in hand. Although some studies in de novo PD patients have shown that sleep quality is not impaired while daytime sleepiness is noticeable^48^, other studies have reported a correlation between sleep and wakefulness disturbances in PD patients^49,50^. Interestingly, one study also reported a decrease in delta power during non-REM sleep in de novo and drug-naïve PD patients^51^, a sleep disturbance similar to that observed during the presymptomatic state in our study.

In NHPs with a stable parkinsonian syndrome, we observed drastic and significant changes during both nighttime and daytime. Indeed, during the night, we observed a significant decrease in N3 and REM sleep, a significant increase in WASO and a dramatic decrease in sleep efficiency, as already described^28,29^ and observed in patients with advanced PD^52,53^. RBDs are among the most common sleep disorders in PD; in RBDs, REM sleep parasomnias lead to abnormal vocalizations and behavior^54^. In our study, no abnormal or violent behavior was observed, consistent with other NHPs studies^28,29^. However, we described a significant loss of muscular atonia during REM sleep in parkinsonian animals, similar to that observed in marmosets^55^ and PD patients^56^. Even if this event is less marked in NHPs than in humans, it suggests an alteration of mechanisms involved in atonia^57^. Dopamine dysregulation can modify the function of brainstem nuclei, such as the locus coeruleus alpha or the pedunculopontine nucleus which are closely connected to the substantia nigra and regulate atonia during REM sleep^54^. As mentioned above, the MPTP dosage used in our study was very progressive, even more than that used in most studies using slow chronic induction^58^. These studies have shown that a slow and chronic MPTP regimen induces a stable parkinsonian syndrome over time, without recovery phenomena; this MPTP regimen also induces damage to multiple brain systems. Thus, we assume that our protocol affected several brain regions, as we observed in synucleinopathy. Given the loss of atonia during REM sleep, we assume that the systems responsible for the decrease in motor neuron excitability, inducing muscle atonia, were affected by the dosage used in our study.

During the day, once a stable parkinsonian syndrome was established, it became difficult to distinguish between naps, which were diffused throughout the day. This finding is consistent with the state of permanent EDS reported by parkinsonian patients. The onset of an increasingly early morning nap is reminiscent of a delayed phase syndrome, which is also reported in patients with PD^59^. Dopamine depletion could also induce disturbances of the circadian rhythm, which is mediated by a regulatory mechanism called process C^60^. Indeed, this link has already been reported in MPTP-NHPs, but more as an inability of the circadian clock to efficiently drive rhythmic locomotor activity^26^. All these observations, especially the early onset of morning naps while sleep efficiency remains constant during the presymptomatic state, are consistent with human studies concluding that EDS may be an NMS unrelated to other sleep disorders^3,61^. Furthermore, several studies have demonstrated a correlation between EDS and dopaminergic treatment^48,62^. In our study, we did not administer dopaminergic treatment, which indicates that EDS in MPTP-treated NHPs is not necessarily associated with drugs and may be due to the early dysregulation of the dopaminergic and other systems.

We acknowledge the limitations of translating experimental animal data to human conditions. Indeed, the use of animal models cannot perfectly mimic the human disease. However, our progressive MPTP-treated NHP model exhibit SWDs, characterized by various objective measures and very similar to those observed in PD patients. Even if this study provides a solid documentation of the clinical evolution of motor symptoms and NMSs, we are aware that this study does not allow us to follow the degeneration of different brain structures; therefore, we cannot definitively conclude about the involvement of one or more neurotransmitters. However, histological studies of brain tissue from MPTP-treated monkeys under differing treatment regimens have shown various forms of brain damages^58^. Indeed, the animals that received acute treatment showed only a dopaminergic lesion^63,64^, whereas the animals that received chronic treatment, which can be considered similar to our protocol, showed damage to the noradrenergic^65^, serotoninergic^66^ and cholinergic^67^ (in older monkeys only) systems in addition to damage to the dopaminergic system. Finally, we assume that only long-term chronic administration of MPTP induced progressive neurodegeneration of other systems that significantly impact the sleep/wake behavior. Further studies are needed to understand whether early and small reductions in dopamine can alter the function of structures involved in sleep/wake behavior or whether other neurotransmitters are directly involved. Nevertheless, this MPTP regimen offers a relevant model of disease progression that can be used as a basis for the investigation of the neuropathological mechanisms of PD and can help in the management of human pathology by delineating a therapeutic window for the development of early neuroprotective strategies. The strength of this study lies in the demonstration of prodromal SWDs in the progressive NHP model of PD, expressed by an alteration of nighttime sleep and daytime nap. This finding may guide the development of EEG biomarkers of PD consisting of SWDs in humans.

## Materials and methods

### Animals

In accordance with the policy of Grenoble Alpes University and the Grenoble Institut of Neurosciences (B3851610008) and with French legislation, experiments were performed in compliance with the European Community Council Directive of 2010 (2010/63/UE) for care of laboratory animals and the ARRIVE guidelines. All procedures were reviewed and validated by the “Comité éthique du GIN n˚004” and was authorized by the Direction Départementale des Services Vétérinaires de l’Isère—Ministère de l’Agriculture et de la Pêche, France. We used three adult male macaques (*Macaca fascicularis*—Mauritius) M1 (8 kg; 8 years old), M2 (10 kg; 10 years old) and M3 (6 kg; 5 years old). Animals were kept under controlled conditions, 12 h light/dark cycles [lights OFF at 19:00], 23 ± 2 °C, and 50 ± 5% humidity. Animals were pair housed, had access ad libitum to food and water and supplemental fresh fruit was given once a day.

### Apparatus

**Figure 5:**
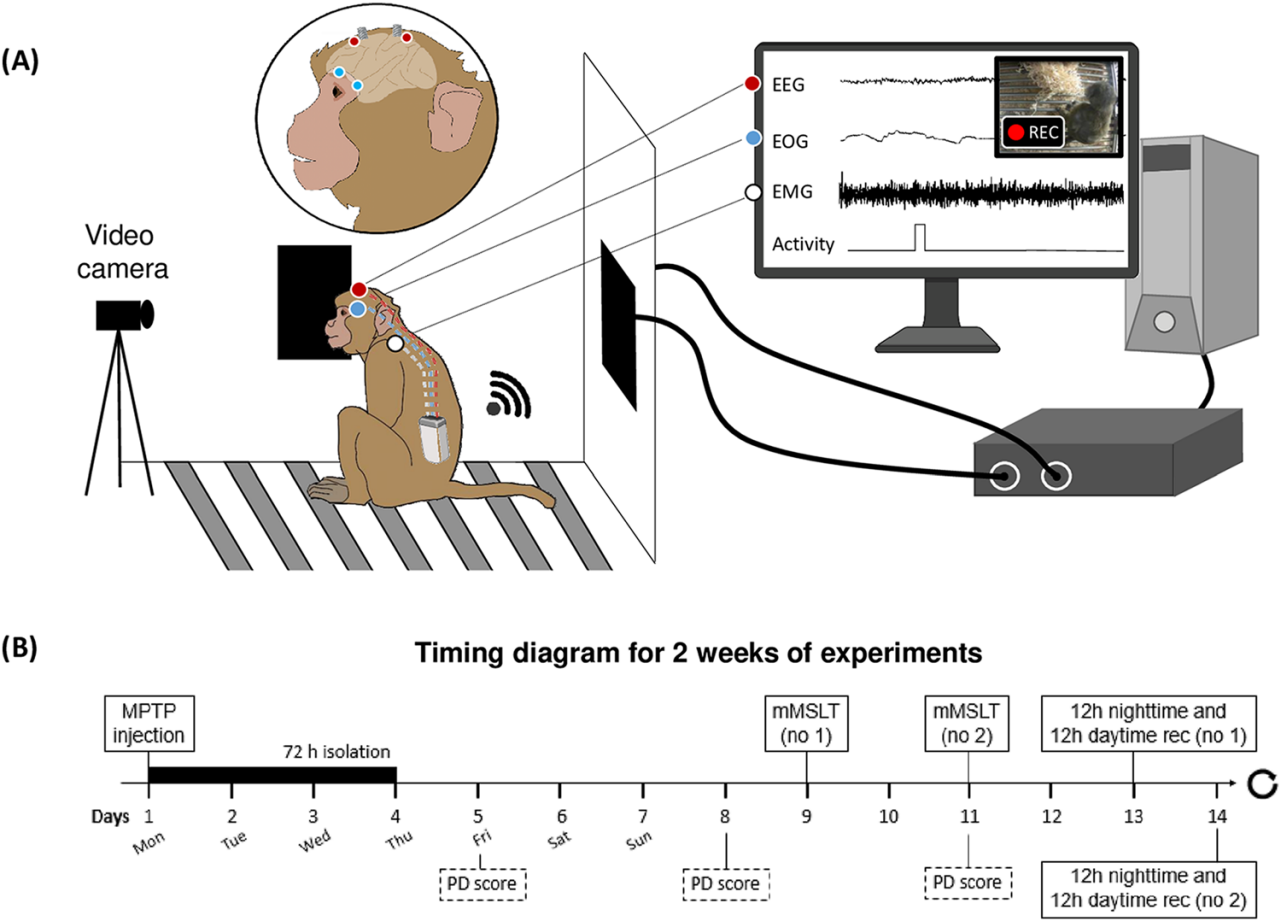
Experimental design. (**A**) Schematic representation of the polysomnographic setup for an implanted animal with the recordings of the brain activity (EEG, in red), the ocular activity (EOG, in blue) and the muscle activity (EMG, in white). Two cage-mounted receivers (black squares) are connected to a data exchange matrix, connected to a computer for data storage and off-line analysis. All recordings are synchronized with a video system. (**B**) Timing diagram for 2 weeks of typical experiments.

Monkeys were implanted with a polysomnographic equipment, a radio-telemeter transmitter (D70-EEE, Data Science International, France) for long-term recording in freely moving animals. The transmitter had three channels biopotential for recording electroencephalography (EEG), electro-oculography (EOG), and electromyography (EMG) signals with a sampling rate of 500 Hz and a gain of 75 and actimetry count. An incorporated magnetically activated switch enabled the implanted transmitter to be switched on and off externally. Signals were acquired via two receivers mounted on the home cage and behavioral cage and then forwarded to a data exchange matrix connected to a computer for data storage and off-line analysis (Fig. 5A).

### Surgery

The surgery was performed under aseptic conditions and general anesthesia. Animals were anesthetized with Ketamine (7 mg/kg, i.m.) and Xylazine (0.6 mg/kg, i.m.) then intubated and switched to isoflurane mixed with oxygen. Animals were spontaneously breathing. Respiration rate, Et-CO_2_ and O_2_ saturation were monitored with a Comdek MD-660P monitor. Saline solution (NaCl 0.9%) was infused intravenously all along the surgery for drug access and hydration. Analgesic therapy (Ketoprofen 2 mg/kg i.m.) were provided during 1-week post-operative period. As it has already been done and described^28,29^, the radio-telemeter transmitter was implanted within a subcutaneous pocket in their back and the electrode leads were tunneled to the skull. EEG was recorded using two electrodes screwed unilaterally (one frontal and one parietal) into the skull, EOG was acquired from two electrodes affixed unilaterally at the orbital arch bone (one at the top and one at the external side) and EMG was monitored from two leads sutured into the neck musculature. The reference was fixed on the skull at the left occipital level.

### MPTP treatment

Monkeys were intoxicated by intramuscular injections of MPTP under light anesthesia (Ketamine 2–4 mg/kg). A progressive protocol was used to obtain a presymptomatic phase (i.e. before the appearance of significant motor symptoms) consisting in small doses of MPTP (0.2–0.5 mg/kg, in NaCl 0.9%) at two-weeks interval until the parkinsonian symptoms were stable. MPTP injections were given significantly less frequently than most other studies^28,29,68^. The protocol was designed to induce very slow degeneration whilst permitting a sufficient number of polysomnographic recordings (Fig. 5B). After each injection of MPTP, we assessed motor score, and the next dose was either similar or adjusted according to the clinical signs observed. M1 received 18 injections (7.55 mg/kg total), M2 received 8 injections (2.2 mg/kg total) and M3 received 18 injections (6.1 mg/kg total). All animals had comparable state of stable parkinsonism.

### Motor score

The severity of parkinsonism was evaluated before, during and after MPTP intoxication in the home cage, using a rating scale, combining the most recurring items from eight commonly used parkinsonism scales^69^. This scale includes eight clinical symptoms (general activity, frequency of each arms movements, posture, bradykinesia, tremor, feeding, freezing and vocalization), rated between 0 (normal) and 2–3 (maximal disability), with a total score out of 25. Evaluations were performed by the same observer 5, 8 and 11 days after the injection at 2 pm for 15 min (Fig. 5B). The spontaneous activity was quantified by the implanted radio-telemeter transmitter and expressed in counts/min. Activity values are collected every 10 s, according to the changes of the signal perceived by the receivers mounted in the cage. If the animal is stationary the signal strength remains constant and equal to zero and if the animal moves the signal strength changes and is counted as movement.

### DaTscan

In addition to this clinical motor evaluation, the first animal (M1) had an individual follow-up of the striatal dopaminergic system, all along the induction of the parkinsonian syndrome, which gives a global idea of the impact of MPTP on the dopamine depletion. These imaging examinations were performed during the healthy state (before the 1^st^ MPTP injection), during the presymptomatic state (after the 2nd and 9th MPTP injection) and finally in stable symptomatic state (after the 18th injection). The fixation of the Ioflupane (^123^I) tracer, a radioligand with a high affinity for presynaptic dopamine transporters, was quantified in the striatum to observe the dopamine depletion. For all the exams, the animal was anesthetized and a dose of 90 MBq was injected intravenously 3 h before the acquisition. Then, the images which had the highest radioactivity count, and the one above and below (three cuts) were selected and averaged. Regions of interest were drawn manually for each striatum and an area of occipital cortex served as a reference for the background noise (region of non-specific ^123^I uptake). The final activity count was determined as the ratio of striatal activity to occipital activity.

### Sleep data analysis

**Figure 6:**
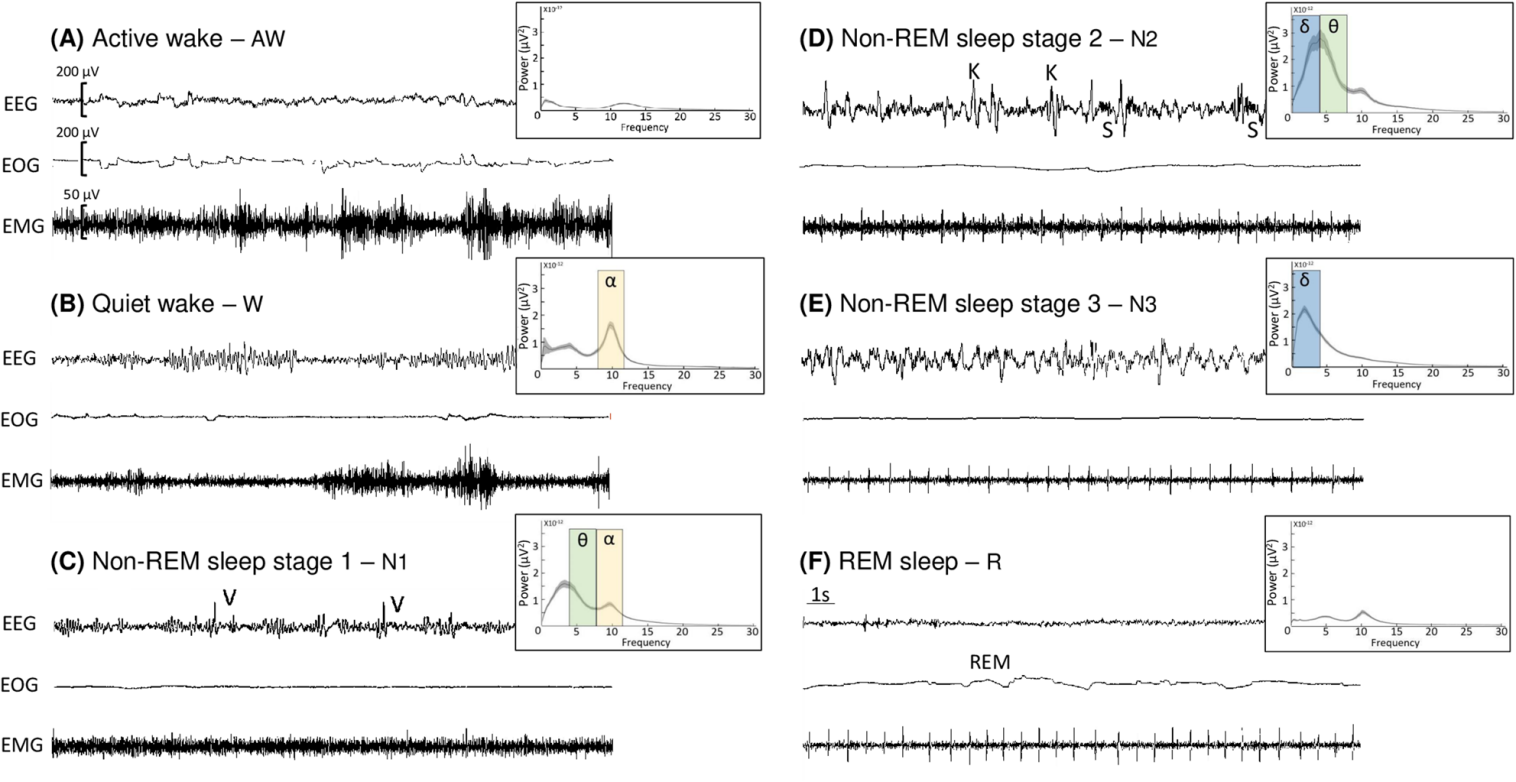
Polysomnographic recordings for sleep/wake stages analysis. 30 s epochs showing (**A**) active wake state with fast and low-voltage electroencephalogram (EEG), high-amplitude electro-oculogram (EOG) and high electromyogram (EMG) activity, (**B**) quiet wake state with alpha waves (8–12 Hz) on EEG, (**C**) light sleep N1 with mix-frequency (alpha and theta waves) and vertex sharp waves (V) on EEG and reduced muscle tones (**D**) light sleep N2 with predominant theta waves (4–8 Hz), spindles (S) and K complex (K) on EEG, absence of EOG and regular muscle tone, (**E**) deep sleep N3 with ample delta frequencies (0.3–4 Hz) on EEG and reduced muscle tone, and (**F**) REM sleep with sawtooth EEG activity, rapid-eyes movements (REM) on EOG and absence of muscle tone. Mean ± 95% confidence interval of the absolute power (µV^2^) of the EEG during each wake and sleep stages is shown to the right of EEG signals.

Sleep scoring was performed offline on a software (NeuroScore, Data Science international). EEG and EOG was bandpass-filtered in the range of 0.3 to 35 Hz and EMG was bandpass-filtered in the range of 10 to 100 Hz. Sleep/wake stages were manually determined according to the American Academy of Sleep Medicine criteria and performed in 30 s epoch^70^. Different stages were identified: active wake (A), quiet wake (W), and non-REM sleep: light sleep stage 1 (N1) and stage 2 (N2), deep sleep stage 3 (N3) and REM sleep (R) (Fig. 6). Movement and chewing artefacts were mostly produced during active wake and were correlated with the simultaneous video observation. EEG power spectral analyses were performed for all scored 30 s epochs, and for the different stages of sleep and wake, to verify whether the visual scoring matched with the expected frequency bands (Fig. 6). All epochs with artefacts were excluded from spectral analysis.

### EMG and EEG spectral analysis

Absolute power spectral density analysis was performed on EMG and EEG signals during active wake during healthy, presymptomatic, and symptomatic states to help the characterization of these different key periods. For this part, 300 samples of random 30 s epochs (100 samples for each animal) of EEG and EMG during active wake and in the different conditions were analyzed. The similarities between the animals allowed us to pool all the data. Then, absolute power spectral density was performed on EMG during REM sleep and on EEG during deep sleep N3 during healthy, presymptomatic, and symptomatic states to observe any changes between these different conditions. For this part, the whole samples of 30 s of N3 or REM sleep, during the different conditions and for each individual animal, were analyzed. In all cases, for each 30 s epoch, an estimate of the power spectral density was computed using the Fast Fourier Transform and Hamming windowing technique (5 s with 50% overlap) (Matlab) and averaged for the final absolute power (µV^2^). Data are expressed in mean ± 95% confidence interval.

### Nighttime sleep quality evaluation

Nighttime sleep data were analyzed from 7 p.m. to 7 a.m. Two 12 h recording sessions per week were performed until a minimum of ten recordings were reached, in each experimental condition; healthy, presymptomatic and symptomatic states. These recordings were made on weekends to be sure to obtain spontaneous behavior, limiting external factors such as noise and the passage of users. Thus, the post-MPTP nighttime recordings were collected 13 and 14 days after the MPTP injection (Fig. 5B). For each 12 h period of nighttime, the sleep latency (SL) (min), total sleep time (TST) (min), duration of each stage (expressed as % of total scoring time), duration of N3 expressed as % of the TST, wake time after sleep onset (WASO) (min), sleep efficiency (expressed in %, as the ratio of TST to the sleep period time^71^) and number of transitions between wake and sleep stages per hour were calculated and averaged within the same disease condition.

### Daytime hypersomnia evaluation

Assessment of hypersomnia was done by long-term recording from 7 a.m. to 7 p.m. Two 12 h recording sessions per week were performed until a minimum of ten recordings were reached, in each experimental condition; healthy, presymptomatic and symptomatic states. These recordings were made on weekends to be sure to obtain spontaneous behavior, limiting external factors such as noise and the passage of users. Thus, the post-MPTP daytime recordings were collected 13 and 14 days after the MPTP injection (Fig. 5B). For each 12 h period of daytime, the first sleep after wake (min), TST (min), duration of each stage (expressed as % of total scoring time) and number of transitions between wake and sleep stages per hour were calculated and averaged within the same disease condition.

### Daytime sleepiness evaluation

Daytime sleepiness was evaluated using a modified multiple sleep latency test (mMSLT), performed 2 h after waking up. In quiet room, the lights were turned off 3 times for a duration of 20 min, 1 h apart (lights-OFF sessions at 10 a.m., 11 a.m. and 12 a.m.). mMSLT were performed twice a week during healthy state until a minimum of ten experimentations were acquired and all along the parkinsonian syndrome induction for a minimum of ten experimentations during presymptomatic and symptomatic states. During these two last conditions mMSLT were performed 9 and 11 days after the MPTP injection to be in sufficient margin of the ketamine injection (Fig. 5B). SL was determined if a 30 s epoch of scorable sleep was observed. If no sleep onset was observed, SL was designated to be 20 min as used in human and NHP studies^72,73^. The two wake stages A and W were pooled together and the two light sleep stages N1 and N2 were pooled together. The following parameters were calculated for each 20 min period of lights-OFF session: SL (min), duration of wake and sleep (min) and averaged by lights-OFF session or for all lights-OFF.

### REM sleep heart rate variability analysis

12 epochs of 2 min of EMG during REM sleep were randomly pick up including the perception of the cardiac R peak which gives us an electrocardiogram-like. For each epoch, beat-to-beat (RR) variability was analysed in the time domain including the mean RR (meanNN) interval and its standard deviation (StdNN) and the coefficient of variation of RR intervals |[StdNN/MeanNN] × 100| showing the relative extent of the data. Poincare plot, which portrays the relationship between successive RR intervals (RR_n+1_ interval plotted against the preceding one RR_n_) was done. This plot provides summary information about RR interval and density distribution which are considered as general measures of heart rate variability.

### Statistical analysis

#### Effect of disease progression on sleep/wake parameters

Standard statistical methods using GraphPad Prism 9 were applied for all data comparisons. On each animal, values were collected randomly during the different time periods corresponding to different states (healthy, presymptomatic, symptomatic). These different states depended on the motor impact produced by the progressive administration of MPTP on the animal. Thus, the data for each state were not acquired longitudinally and consequently could not be paired with each other. Therefore, after testing the normal distribution of the data with a Shapiro test, a one-way non-parametric ANOVA; Kruskal–Wallis test, followed by Dunn's multiple comparisons; was applied for all individual sleep or wake parameters reported in Tables 1 and 2 and for the transitions between sleep and wake stages during both nighttime and daytime according to the state of the disease: during healthy, presymptomatic and symptomatic states, for each individual animal. Data are presented as mean ± standard error of the mean (SEM) and the statistical significance was considered at a probability (p) value ≤ 0.05.

#### Effect of disease progression on signal power of EEG and EMG

Standard statistical analyses were done with MatLab, using a one-way ANOVA test to compare the signal power, per 1 Hz increment, according to the state of the disease: during healthy, presymptomatic and symptomatic states, for each individual animal. The different spectral analyses are expressed as mean ± 95% confidence interval and the statistical significance was considered at a probability (p) value ≤ 0.05. It should be noted that ANOVA is generally robust to violations of the normality assumption, which we did not test for this part of the study.

#### Effect of MPTP on dopaminergic cells

A Mann–Whitney rank-sum test was performed to compare the loss of TH neurons between all MPTP-treated animals and a control animal for three regions of interest: putamen, caudate and SNc.

## Supplementary Information

Supplementary Table 1.
