# Supplementary material for: Early onset of sleep/wake disturbances in a progressive macaque model of Parkinson’s disease
Source: Sci Rep. 2022 Oct 19;12:17499. doi: 10.1038/s41598-022-22381-z (PMC9581909; doi:10.1038/s41598-022-22381-z)
Supplement: Supplementary file 1 — Supplementary Table 1. [file 41598_2022_22381_MOESM1_ESM.pdf]

Early onset of sleep/wake disturbances in a progressive macaque model of Parkinson’s disease

Aurélie Davin, PhD[1,2], Stéphan Chabardès, PhD, MD[2,3], Hayat Belaid, PhD[4], Daniel Fagret, MD[5], Loic Djaileb, MD[5], Yves Dauvilliers, PhD[6], Olivier David, PhD[2,7], Napoléon Torres-Martinez, PhD[1], Brigitte Piallat*, PhD[2]

		Injection number	Cumulative dose of MPTP (mg/kg)	Score of parkinsonism (mean ± SEM)	General activity (0-3)	Frequency of arms movement (0-3 for each arm) LEFT	RIGHT	Flexed posture (0-3)	Bradykinesia (0-3)	Tremor * (0-3)	Eating (0-3)	Freezing (0-2)	Vocalization (0-2)
**M1 8 y.o. 8 Kg**	Presymptomatic	1 → 10	3.9	3 ± 0.3	1.3 ± 0.1	0.7 ± 0.1	1.1 ± 0.3	0	0	0	0	0	0
	Syndrome stabilization	11 → 18	7.55	9.1 ± 1.1	1.7 ± 0.5	1 ± 0.1	1.1 ± 0.1	2 ± 1.0	1.6 ± 0.5	0.9 ± 0.8	0	0.8 ± 0.5	0
	Symptomatic (stable)	18	7.55	13.8 ± 0.2	**2 ± 0**	**1.3 ± 0.2**	**1.8 ± 0.1**	**3 ± 0**	**2 ± 0**	**1.1 ± 0.2**	0	**1.4 ± 0.1**	**1.2 ± 0.1**
**M2 10 y.o. 10 Kg**	Presymptomatic	1 → 6	1.5	4.6 ± 0.1	1.5 ± 0.1	2.3 ± 0.2	0	0	0.6 ± 0.1	0	0	0	0
	Syndrome stabilization	7 → 8	2.2	15.4 ± 1.6	2.8 ± 0.5	2.5 ± 0.2	1.7 ± 0.1	2.8 ± 1.1	1.6 ± 0.5	0.9 ± 0.5	1.4 ± 0.1	0.7 ± 0.5	1 ± 0.5
	Symptomatic (stable)	8	2.2	18.9 ± 0.1	**3 ± 0**	**3 ± 0**	**2 ± 0**	**3 ± 0**	**2 ± 0**	**2.6 ± 0.1**	**0.5 ± 0.1**	**1.7 ± 0.2**	**1.1 ± 0.1**
**M3 6 y.o. 5 Kg**	Presymptomatic	1 → 12	5.5	2.8 ± 0.1	1.6 ± 0.1	0	0	0	0	0	1.2 ± 0.1	0	0
	Syndrome stabilization	13 → 18	6.1	9.8 ± 1.3	2.1 ± 0.6	0.8 ± 0.1	1.2 ± 0.5	2.1 ± 1.0	2 ± 0.8	0	0.8 ± 0.1	0.8 ± 0.5	0
	Symptomatic (stable)	18	6.1	16.4 ± 0.3	**3 ± 0**	**1.8 ± 0.2**	**2.4 ± 0.1**	**3 ± 0**	**2 ± 0**	**1.8 ± 0.3**	**1 ± 0.1**	**1.4 ± 0.1**	0

*mainly action tremor; only M2 experienced resting tremor

Supplementary Table 1: Mean values and standard deviations of each clinical item of the parkinsonian scale used during the different disease states: presymptomatic state, syndrome stabilization and stable symptomatic state. Each item is scored between 0 (no difference with healthy state) and 2 or 3 (maximum disability compared to healthy state), with a total score out of 25.
